# Effect of Reference Scheme on Power and Phase of the Local Field Potential

**DOI:** 10.1162/NECO_a_00827

**Published:** 2016-03-04

**Authors:** Vinay Shirhatti, Ayon Borthakur, Supratim Ray

**Affiliations:** Centre for Neuroscience, Indian Institute of Science, Bangalore, India, 560012

## Abstract

Brain signals are often analyzed in the spectral domain, where the power spectral density (PSD) and phase differences and consistency can reveal important information about the network. However, for proper interpretation, it is important to know whether these measures depend on stimulus/behavioral conditions or the reference scheme used to analyze data. We recorded local field potential (LFP) from an array of microelectrodes chronically implanted in area V1 of monkeys under different stimulus/behavioral conditions and computed PSD slopes, coherence, and phase difference between LFPs as a function of frequency and interelectrode distance while using four reference schemes: single wire, average, bipolar, and current source density. PSD slopes were dependent on reference scheme at low frequencies (below 200 Hz) but became invariant at higher frequencies. Average phase differences between sites also depended critically on referencing, switching from 0 degrees for single-wire to 180 degrees for average reference. Results were consistent across different stimulus/behavioral conditions. We were able to account for these results based on the coherence profile across sites and properties of the spectral estimator. Our results show that using different reference schemes can have drastic effects on phase differences and PSD slopes and therefore must be interpreted carefully to gain insights about network properties.

## 1 Introduction

Local field potential (LFP) recorded using microelectrodes implanted inside the brain is thought to reflect mainly the overall synaptic activity of the neuronal population (Buzsáki, Anastassiou, & Koch, 2012; Einevoll, Kayser, Logothetis, & Panzeri, 2013; Logothetis, 2003; Mitzdorf, 1985; Nunez & Srinivasan, 2006) and provides clues about the properties of the neuronal network around the microelectrode. LFPs are usually studied in spectral domain by computing the Fourier transform of its autocorrelation function, called the power spectral density (PSD), in which brain rhythms associated with different behavioral states (Buzsaki, 2006; Buzsáki & Draguhn, 2004) are captured as band-limited peaks. In addition, PSDs of brain signals have a typical 1/f form, whose slope reveals important information about the neuronal network, such as the nature of noise. For example, while white noise produces a slope of zero, a slope of 2 can be generated by shot (Brownian) noise, whose origin might be due to up-down states of slow-wave sleep just as a telegraphic process or from an exponential relaxation process of synaptic currents that is driven by random spiking (Baranauskas et al., 2012; Bédard, Kröger, & Destexhe, 2006a; Miller, Sorensen, Ojemann, & Nijs, 2009; Milstein, Mormann, Fried, & Koch, 2009). Additionally, filtering properties of the network, such as capacitive coupling or filtering by active conductances, are captured in the slope (Bédard et al., 2006a; Bédard & Destexhe, 2009; Lindén, Pettersen, & Einevoll, 2010; Logothetis, Kayser, & Oeltermann, 2007). Further, while earlier studies mainly focused on the power of the signal at different frequencies (captured using the PSD), recent theories have proposed a potential role of phase in cortical processing. For example, communication between two brain areas can be facilitated by aligning their relative phases appropriately (communication through coherence hypothesis; Fries, 2005; Womelsdorf et al., 2007). Testing these hypotheses require an accurate estimation of the power and phase of the LFP at multiple sites.

Potential recorded at the microelectrode tip is relative to some reference voltage, the choice of which can potentially change the properties of the signal (Nunez & Srinivasan, 2006). LFPs are usually measured relative to a single electrode or wire placed far away from the microelectrode (called a *single-wire reference* here). However, a critical issue with this scheme is that if the reference wire itself picks up some neural activity, all microelectrodes show that activity as well (because the reference signal is subtracted from the potential obtained from each microelectrode). Further, sometimes all the electrodes pick up some common noise, and it is desirable to remove this common component and focus only on the local neural activity specific to the location near the electrode. This is achieved by constructing another reference signal that represents the common noise and subtracting it from the recording signal of interest. Some of the common referencing schemes (proposed mainly for EEG data analysis, but the same extends to LFP analysis as well) are average reference (the reference signal is the average of all electrodes), bipolar reference (each electrode is referenced to a nearby electrode), and current source density (CSD: the reference signal for a 2D grid is the average of four nearest neighbors). The reference signal comes progressively closer to the recording signal as we move from average reference, bipolar to CSD, highlighting progressively localized neural activity. Note that we use the term *reference* for bipolar and CSD schemes also, even though they involve a single or up to four electrodes only (in this letter, we only use a 2-dimensional CSD reference because we do not have recordings along the depth of the cortical tissue; see equation 2.1).

There is no gold standard when it comes to the choice of a referencing scheme (Nunez & Srinivasan, 2006; Schiff, 2005). Instead the choice is often arbitrary, depending on the preference of the experimenter, the level of common noise, and the specific question of interest. Previous studies using EEG or electrocorticogram (ECoG) signals have shown that phase consistency between electrode pairs when single-wire reference is used depends on the amplitude of the reference signal itself (Fein, Raz, Brown, & Merrin, 1988; Hu, Stead, Gardner, & Worrell, 2007; Hu, Stead, Dai, & Worrell, 2010; Nunez & Srinivasan, 2006; Schiff, 2005). However, the effect of different reference schemes on PSD slopes and phase differences or consistency has not been well studied, especially in LFP data. We therefore recorded LFP from chronically implanted 10 × 10 microelectrode arrays in monkeys and compared PSD slopes and phase relationships under the referencing schemes mentioned above. Further, we tested whether PSD slopes and phase relationships depended on stimulus conditions or the attentional state of the animals.

## 2 Materials and Methods

All the experiments carried out were in adherence to the protocols approved by the Institutional Animal Care and Use Committee of Harvard Medical School. Behavioral task, data collection procedure, and electrode selection criteria are the same as in previous studies (Ray & Maunsell, 2010, 2011b) and some details are omitted here. Briefly, LFP signals were recorded from two male rhesus monkeys (*Macaca mulatta*) using a 10 × 10 microelectrode array (Blackrock Microsystems, 96 active electrodes) implanted in area V1 of the right cerebral hemisphere (about 15 mm anterior from the occipital ridge and 15 mm from the midline; the corresponding receptive fields were in the lower left quadrant spanning about 2 × 2 degrees of visual angle at an eccentricity of about 4 degrees). Raw data were filtered between 0.3 Hz (Butterworth filter, first order, analog; integrated in the recording hardware) and 500 Hz (Butterworth, fourth order, digital) and digitized at 2 kHz (16 bit resolution). The LFP signals were originally referenced with respect to a single wire placed on the dura near the electrode grid (this is an insulated wire that is typically stripped by 1 to 2 cm to expose the metal and placed under or on the dura within a few centimeters of the recording array). Only electrodes conforming to reliable estimation of the receptive field center (SD less than 0.1 degree across days, mapped by flashing small Gabor stimuli on a rectangular grid that spanned the receptive fields of all the electrodes) were used, yielding 27 and 62 electrodes from monkeys 1 and 2, respectively (one region of the array implanted in monkey 1 did not yield usable signals). The monkeys performed an orientation-change detection task (monkey 1, 10 sessions, and monkey 2, 17 sessions). They maintained fixation within 1 degree of a small central dot located at the center of a CRT video display (100 Hz refresh rate, 1280 × 768 pixels, gamma corrected), while two achromatic, odd-symmetric Gabor stimuli were synchronously flashed for 400 ms with an average interstimulus period of 600 ms. One of the Gabors was centered on the receptive field of one of the recording sites (new location for each session), while the other was on the opposite side of the fixation point at an equal eccentricity. The monkey was cued to attend to one of them in blocks of trials and rewarded for detecting a change in the orientation (by 90 degrees) in one of the presentations. Both the stimuli were static with an SD of 0.5 degree, spatial frequency of 4 cycles per degree (CPD) and at the preferred orientation of the recording site (different for each session). The contrasts of the two stimuli were matched on each presentation and could take one of eight values—0%, 1.6%, 3.1%, 6.2%, 12.5%, 25%, 50%, and 100%—chosen pseudo-randomly. On average, each contrast was repeated 79 times (range, 55–101) for monkey 1 and 74 times (range, 47–120) for monkey 2, for each attentional condition. Except Figure 2B, all results are shown for the attend-out condition (when the monkey was not attending to the stimulus inside the receptive field).

### 2.1 Reference Schemes

The recordings were initially referenced to a single wire placed on the dura near the microelectrode grid (single-wire reference). For average reference, we took the average of 27 and 62 electrodes for the two monkeys as the reference signal. For computing bipolar reference, we took all pairs of the selected electrodes, yielding 702 (27 × 26) and 3782 (62 × 61) pairs of electrodes with varying interelectrode differences, out of which 351 and 1888 pairs were unique (only one of the two pairs with the same set of electrodes ((x, y) and (y, x)) was used; electrode distances more than 4 mm (0 and 3 pairs for the two monkeys) were discarded). Such a bipolar referenced signal was assumed to be recorded from a virtual electrode located at the middle of the two contributing electrodes. Therefore, for bipolar reference, the nearest electrodes were separated by a distance of 0.2√2 mm (assume three real electrodes at (0, 0), (0, 0.4), and (0.4, 0) mm; the virtual bipolar electrodes would be at (0, 0.2) and (0.2, 0) with a separation of 0.2√2 mm). To avoid any directional bias, bipolar referencing was done using a center-out scheme in which the signal recorded from an electrode farther from the center of the recording array was subtracted from the signal from the nearer one; other schemes (such as subtraction of lateral and dorsal electrodes from medial and ventral ones) yielded similar results for all bipolar pairs that did not share a common electrode. Also, to test whether the inclusion of a single single-wire referenced electrode in the estimation of multiple bipolar electrodes could influence our results, we also constructed a set of bipolar electrodes in which each single-wire electrode was used at most once. The PSDs estimated using this restricted set of bipolar electrodes were indistinguishable from the PSDs obtained from the full set (as shown in Figure 3B).

For a 2D microelectrode array, we defined the CSD referenced voltage at coordinate (*x*, *y*) of a particular electrode as

(2.1) $$\begin{array}{l} \text{CSD}\left(x,y\right)=V\left(x,y\right)\\ \;-\frac{V\left(x-1,\;y\right)+V\left(x+1,\;y\right)+V\left(x,\;y-\;1\right)+V\left(x,\;y+1\right)}{4}, \end{array}$$

that is, the CSDs were computed by subtracting the mean of four neighboring electrodes 400 *μ*m apart. This analysis was limited to electrodes that had four good neighbors (electrodes at the edge of the array or electrodes for which any one neighboring electrode was broken were excluded), yielding 16 and 29 electrodes for monkeys 1 and 2.

### 2.2 Phase Coherence and PSD

If the Fourier coefficients of two signals are expressed as *A_k_ (f)e ^jφ_k_^ (f)* and *B_k_ (f)e ^jθ_k_^ (f)*, (where *f* is the frequency and *k* is the trial number, which varies from 1 to *N*), phase coherence or phase locking value is calculated by (Lachaux, Rodriguez, Martinerie, & Varela, 1999)

(2.2) $$C_{\text{phase}}\left(f\right)=\frac{1}{N}\left|\sum_{k}e^{j\left(\phi_{k}\left(f\right)-\theta_{k}\left(f\right)\right)}\right|.$$

Given *N* phase angles, the angular deviation is defined as

(2.3) $$\sigma_{\text{phase}}=\sqrt{2\left(1-R\right)},$$

where *R* is the length of the mean resultant vector (same as *C_phase_* in equation 2.2 if phase angles correspond to phase differences across trials). This is a measure of the circular spread about the mean resultant vector. Because the angular deviation varies over [0, √2], it is preferred over the standard deviation, which is unbounded for directional statistics (Berens, 2009; Zar, 2010). Circular statistics were performed using CircStat (Berens, 2009).

PSDs, phases, and phase coherence were computed using the multitaper method (Thomson, 1982), implemented in Chronux 2.0 (Mitra & Bokil, 2007), an open source, data analysis toolbox available at http://chronux.org. Briefly, the multitaper method reduces the variance of spectral estimates by premultiplying the data with several orthogonal tapers known as Slepian functions (Jarvis & Mitra, 2001; Mitra & Pesaran, 1999). We used a single taper to maximize the frequency resolution. For baseline analyses (all Figures except Figure 2), data for all eight contrasts were pooled to compute the PSD, and PSDs across sessions (10 and 17 for the two monkeys) were averaged to get one PSD per electrode. For stimulus period and attention analysis (see Figure 2), for each session, we chose only electrodes whose receptive fields were within 0.2 degree of the stimulus center, which yielded 63 and 89 electrodes for the two monkeys across all sessions, out of which 23 and 53 electrodes were unique. As for the baseline case, we averaged PSDs for a unique electrode across sessions to get a single estimate of PSD per electrode.

We corrected the PSDs for the amplifier roll-off, which was experimentally determined by passing a sinusoidal signal at various frequencies through the data recording system and measuring the output (see online supplementary Figure 1). This experimentally determined transfer function was very similar to the theoretical transfer function up to about 500 Hz (calculated based on the properties of the Butterworth filters used in the Blackrock data acquisition system), although at higher frequencies (above about 500 Hz), the experimentally determined function had higher power indicating amplifier or measurement or digitization noise. To avoid any possible influence of the filter roll-off on our results, the slopes are reported only up to 400 Hz. The Blackrock amplifier has an input impedance above 1 T*Ω*, and therefore the amplifier-induced distortion in the phase and amplitude in this frequency range is negligible (Stacey, Kellis, Patel, Greger, & Butson, 2012).

#### 2.2.1 Curve Fitting

We fitted the PSDs with the following function (Miller, Sorensen, Ojemann, & Nijs, 2009):

(2.4) $$P=A.f^{-\alpha }+B,$$

where *P* is the PSD and *f* is the frequency, while *A* (scaling function), *B* (noise floor), and *α* (slope) are free parameters. The parameters were obtained using least square minimization using the program fminsearch in Matlab. Data corresponding to the frequencies of Monitor refresh rate (100 Hz), line noise, and its harmonics were not included in the analysis. Slopes were computed in steps of 10 Hz between 20 and 400 Hz by taking PSD segments of ±15 Hz around each frequency point. Larger fit lengths (±25 or ±50 Hz) resulted in smoothing the slope function (see supplementary Figure 2A), but otherwise the results remained unchanged.

## 3 Results

**Figure 1:**
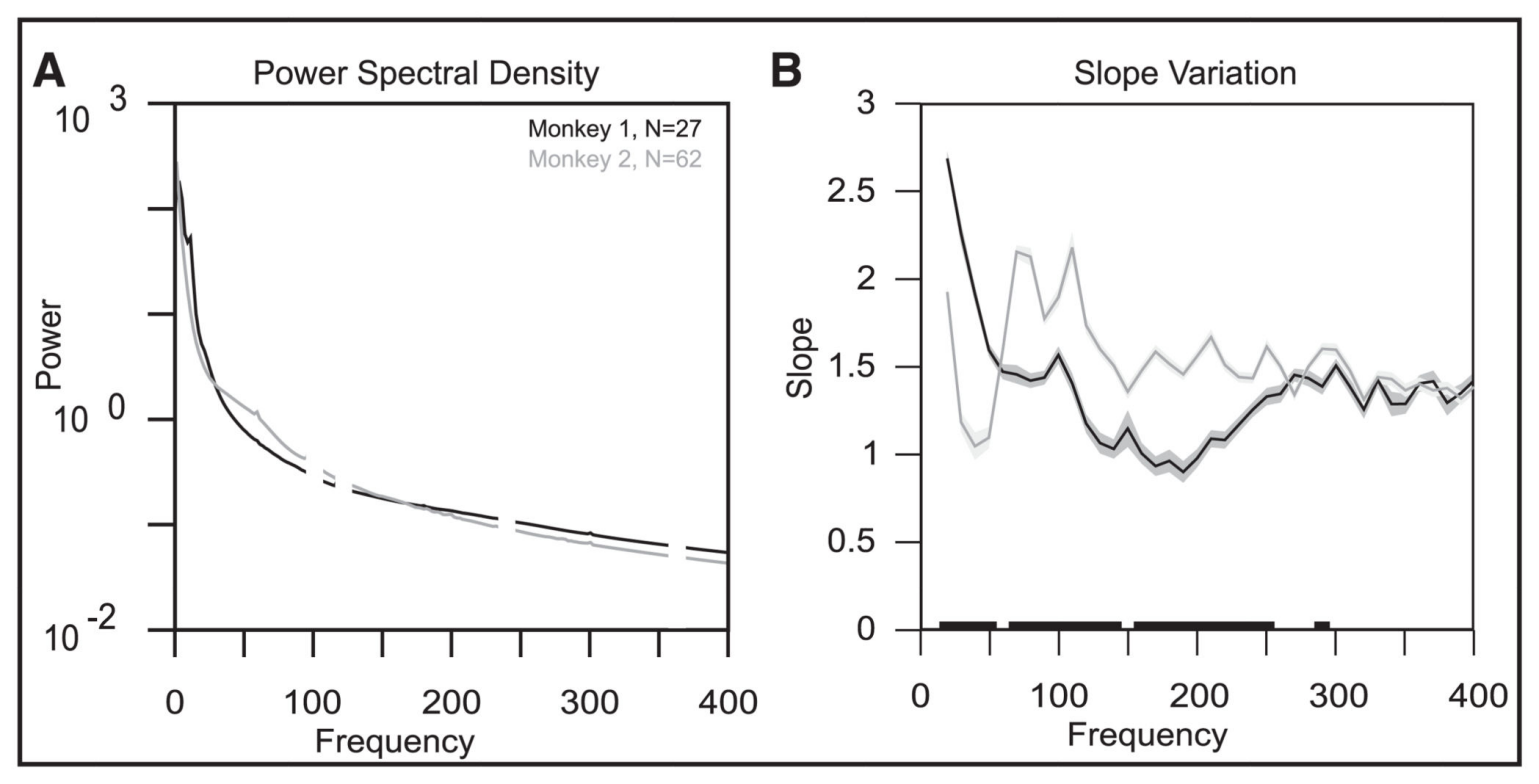
Power spectral density (PSD) slope analysis. (A) Mean PSD across electrodes (denoted by N in the legend) during the baseline period (500 ms to 0 ms interval before stimulus onset) for the two monkeys. Power at frequencies around the monitor refresh rate (100 Hz) and noise harmonics (120, 240, and 360 Hz) has been masked for visual clarity. (B) Mean PSD slope as a function of frequency, computed between 20 and 400 Hz in steps of 10 Hz. The shaded region denotes the SEM of the slope. Black markings on the frequency axis denote frequencies at which the difference in the slopes was statistically significant (ANOVA, *p* < 0.05 with Bonferroni correction).

Figure 1A shows the mean PSD of the single-wire referenced LFP signals across electrodes after amplifier roll-off correction (see supplementary Figure 1 for details) for the baseline period (500 ms to 0 ms before the stimulus onset; attend-out condition) for monkeys 1 and 2. The PSD slopes obtained by fitting equation 2.4 on ±15 Hz segments around each frequency are shown in Figure 1B. LFP power did not decrease with a constant slope with frequency, suggesting that LFPs did not follow a universal power law that extended to a large frequency range. At frequencies below approximately 250 Hz, PSD slopes varied considerably with frequency and also across animals. However, at higher frequencies, the slopes settled to a value of approximately 1.4 for both monkeys. Results were similar when different fit lengths were used (supplementary Figure 2A) or when the PSD was computed over a different time period (supplementary Figures 2B and 2C).

### 3.1 Effect of Stimulus Contrast on PSD Slopes

**Figure 2:**
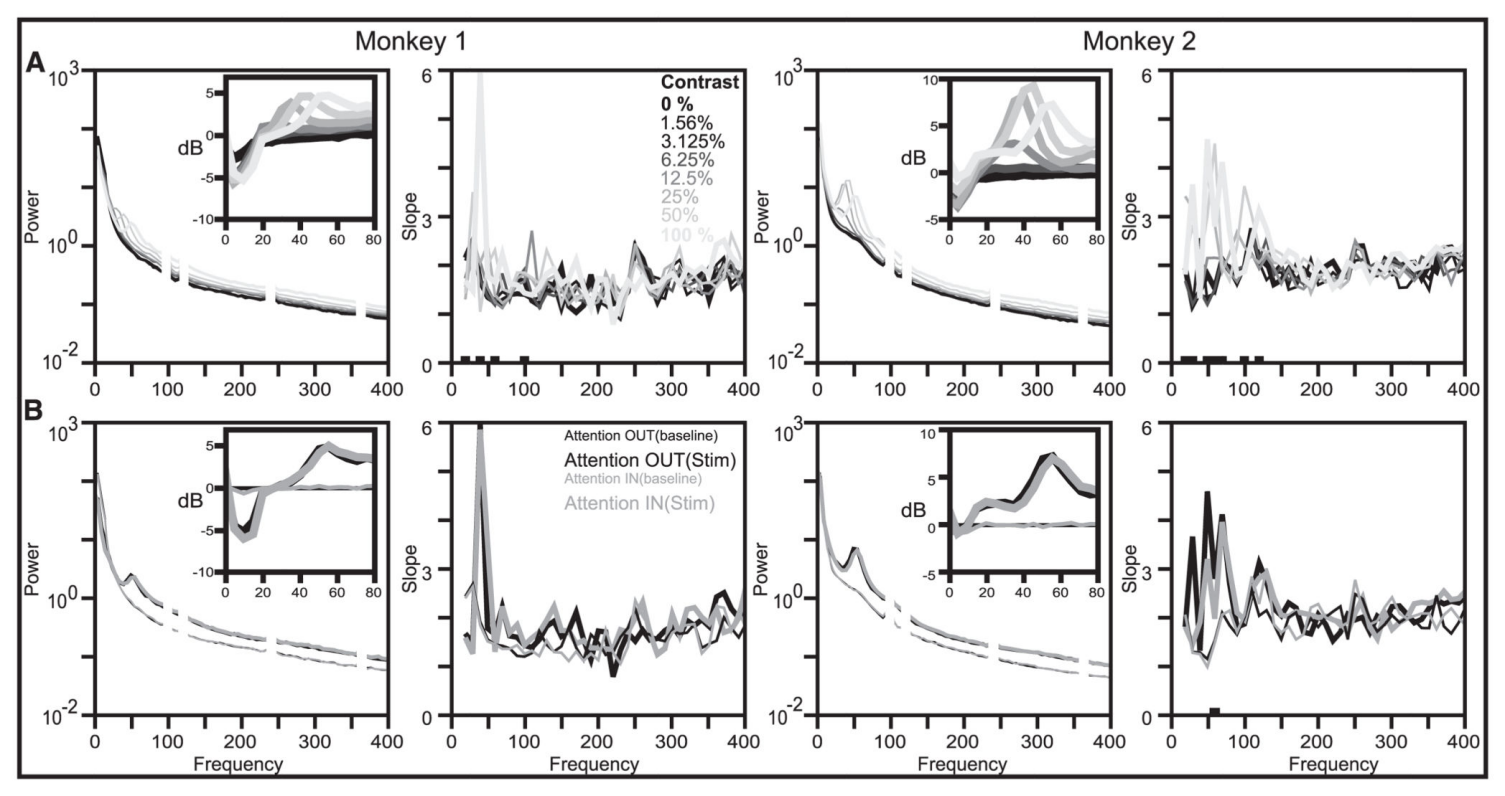
Effect of stimulus contrast and attention on PSDs and slopes. (A) Mean PSD (left) and mean PSD slopes (right) during the stimulus period (200 ms–400 ms after stimulus onset) for different stimulus contrasts (indicated in the second column). The traces corresponding to the lowest (0%; black) and highest contrast (100%, lightest gray) are plotted thicker for clarity. The insets in the first and third columns show the change in power (in decibels) from the baseline period (300–100 ms before stimulus onset) for the different contrast conditions to highlight the suppression of alpha power at about 10 Hz and increase in gamma power above 30 Hz. The black markings over the frequency axis denote the frequencies at which the difference in the slopes is statistically significant (*p* < 0.05; Bonferroni corrected; ANOVA). SEMs are omitted for clarity. (B) Mean PSD and corresponding slopes during the baseline period (300–100 ms before stimulus onset) and stimulus period (200–400 ms after stimulus onset; only 100% contrast condition is shown) when attention was directed inside (Attention IN) or outside (Attention OUT) the receptive field. The insets in the first and third columns show the change in power (in decibels) from the Attention OUT baseline condition.

We next checked whether the PSD slopes depended on stimulus conditions or the behavioral state of the animals. Figure 2A shows the mean PSDs when a Gabor stimulus of varying contrasts was presented and the corresponding slopes. For this analysis, PSDs (of single-wire referenced LFP signals) were computed between 200 and 400 ms after stimulus onset (this time period was chosen to avoid strong stimulus related transients; see Figure 1B of Ray & Maunsell, 2010, for time-frequency power spectrum), and only electrodes that were well stimulated by the stimulus (receptive field centers were within 0.2 degree of the stimulus center; see section 2) were used, yielding 23 and 53 electrodes for the two monkeys. Note that due to the shorter analysis interval of 200 ms, the slopes were noisier (see supplementary Figure 2C). Stimulus onset led to a decrease in power around the alpha band (< 20 Hz) and an increase in the gamma band (about 30–80 Hz) (highlighted in the insets), whose center frequency increased with stimulus contrast (Jia, Xing, & Kohn, 2013; Ray &; Maunsell, 2010). These stimulus-dependent changes in the PSD resulted in occasional differences in slopes across contrasts at low frequencies (< 100 Hz). However, in spite of a clear elevation in power, the slopes remained unchanged with contrast at higher frequencies.

One possible explanation for the invariance of slopes at high frequencies is that the instrument noise floor was higher than physiological neural noise at these frequencies, so the PSD slopes were essentially determined by the statistics of the amplifier or filter noise. However, the absolute power at high frequencies was much higher during the stimulus period than baseline (this increase in high-gamma power is due to the increase in firing rate during the stimulus period; see Ray & Maunsell, 2011a, for a detailed study on the relationship between high-gamma power and multiunit firing rates using data from the same monkeys), and yet the slopes were similar during stimulus and baseline periods, which rules out the possibility of instrumentation noise biasing our results.

### 3.2 Effect of Attention on PSD Slopes

Figure 2B shows the PSDs during baseline (300–100 ms before stimulus onset to match the stimulus analysis period; thin lines) and during the presentation of a stimulus of 100% contrast (200–400 ms after onset) when attention was directed outside (black) or inside (gray) the receptive field. Attention led to well-studied changes in alpha and gamma bands (see the inset), but not at other frequencies. In the alpha range, we observed a significant reduction in power due to attention (monkey 1: reduction of 14.3% and 16.7% for baseline and stimulus periods, *p <* 10^−5^ for both; monkey 2: reduction of 12.9%, *p <* 10^−6^ for baseline period, and 1.2%, *p* = 0.62 for stimulus period, paired *t*-test; note that monkey 2 had very weak alpha, so the effect was not observed at the stimulus period). We also observed a significant increase in gamma center frequency with attention (monkey 1: mean shift of 1.74 Hz, *p* = 0.05; monkey 2: mean shift of 1.60 Hz, *p <* 10^−2^, paired Wilcoxon signed rank test; note that for both monkeys, the analysis interval of 200 ms duration (spectral resolution of 5 Hz) led to noisy estimates of peak frequencies), consistent with prior studies (Bosman et al., 2012). Similar results were obtained for lower-contrast stimuli also (data not shown). Importantly, in spite of clear changes in alpha and gamma bands, there was no change in the slope of the PSD at any frequency range, suggesting that the effect of attention was spectrally localized.

### 3.3 Effect of Reference Scheme on PSD Slopes

**Figure 3:**
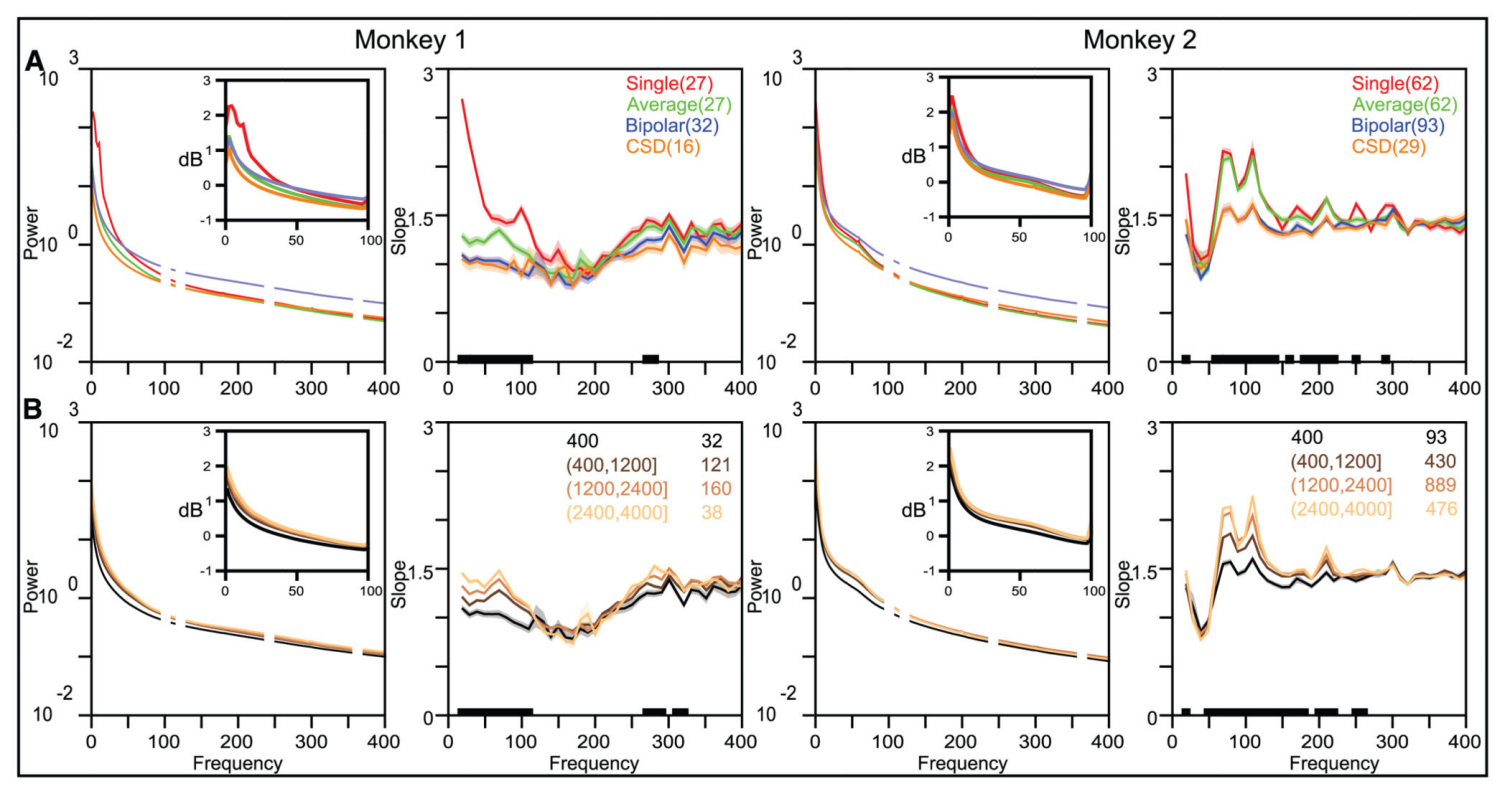
Effect of referencing on PSDs and slopes. (A) Mean PSDs (computed between 500 and 0 ms before stimulus onset; left plot) and the corresponding slopes (right plot) calculated for the single-wire reference (red), average reference (green), bipolar reference (blue), and CSD (orange) for monkeys 1 and 2. The number of electrodes averaged is given in the inset in the first and third columns. (B) Mean PSDs (left plot) and slopes (right plot) using bipolar reference where the reference electrode was taken from varying distances from the recording electrode (distance ranges in *μ*m and the number of electrode pairs are shown in the inset in the first and third columns). The insets in the first and third columns show the PSDs between 0 and 100 Hz, the typical range used in most LFP studies.

Figure 3A represents the mean PSD across electrodes of LFP signals for the baseline period (500 ms–0 ms before the stimulus onset) of monkeys 1 and 2 using four referencing schemes: single-wire reference (red trace), average reference (green trace), bipolar reference (obtained by subtracting the signal from another electrode 400 *μ*m away; blue trace), and CSD (orange trace), and the corresponding slopes.

At low frequencies (< 200 Hz), the slope depended on the reference type: it was significantly larger for single-wire and average references compared to bipolar and CSD references (for which the reference signal was constructed from electrodes that were close to the recording electrode). In contrast, slopes at frequencies beyond 200 Hz varied much less with the referencing scheme. Similar results were obtained when slopes were determined using different frequency fit ranges or when PSDs were computed for a different time period (as in supplementary Figure 2; data not shown).

To study the effect of bipolar referencing in more detail, we chose the reference electrode from progressively larger distances. Figure 3B shows the average PSDs and slopes when the reference electrode was selected from four different distance ranges (shown in the legend along with the number of electrode pairs in each category). Moving the reference electrode away increased the slope at low frequencies for both monkeys but had a negligible effect at frequencies beyond approximately 200 Hz.

### 3.4 Phase Coherence across Electrodes

**Figure 4:**
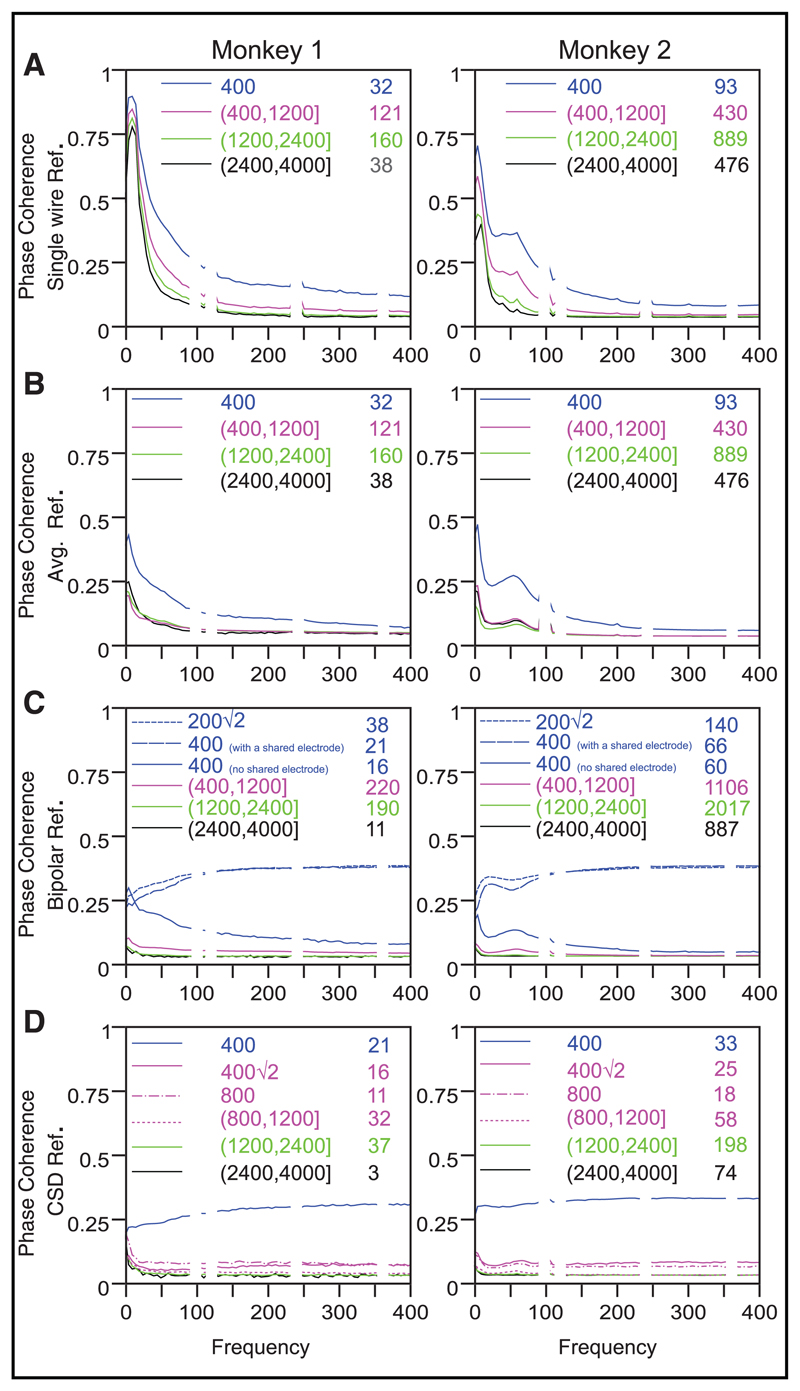
Phase coherence after the signals are referenced using different schemes. The insets show the interelectrode distance ranges and the number of electrode pairs in each range. We chose the baseline period between 300 ms and 100 ms interval before stimulus onset such that the analysis durations were the same for baseline and stimulus conditions (200–400 ms after onset; results are shown in supplementary Figure 4); similar results were obtained if the baseline period was chosen between 500 ms and 0 ms instead. (A) Single-wire reference. (B) Average reference. (C) Bipolar reference. (D) CSD reference. For bipolar and CSD reference schemes, we show some additional ranges (0.2√2 for bipolar, 0.4√2 and 0.8 mm for CSD) at which the computed referenced signals share a common component, which largely determines the coherence and phase differences. For the bipolar reference scheme, we also split the 0.4 mm distance range into two cases—one where the bipolar pairs share a common component and other where they do not.

To explain the changes in PSD slopes with referencing, we first computed the PSDs of the reference signals themselves (see supplementary Figure 3; note that for the bipolar reference, the reference signal is simply the single-wire referenced signal recorded from a neighboring electrode). We found that at low frequencies, the power of all the reference signals was comparable to the single-wire referenced signal, but at higher frequencies, the power of the reference signals was much smaller than the power of the single-wire referenced signal (see Supplementary Figure 3). Because the average reference and the CSD signals are computed by averaging signals from several electrodes, power of this reference signal at a particular frequency depends on the phase relationship between electrodes. Of particular importance is the consistency in the phase difference across time (here, computed for time intervals at a particular position relative to the onset of a stimulus) typically measured using coherence or phase coherence. We have reported phase consistency using several different measures in a previous study (Srinath & Ray, 2014); the results are summarized below and in Figure 4.

We observed that while we were using single-wire reference, the phase coherence between electrodes was high at low frequencies but decreased with increasing frequency and finally approached a constant baseline value above about 100 Hz (see Figure 4A). This baseline value was greater than zero only because of a positive bias in the coherence estimator that depends on the number of trials: using an unbiased estimator such as pairwise phase consistency (Vinck, van Wingerden, Womelsdorf, Fries, & Pennartz, 2010) showed that the true coherence was zero above about 100 Hz (Srinath & Ray, 2014). When other reference schemes were used, phase coherence was reduced to baseline levels at almost all frequencies, which suggested that most of the observed coherence could simply be due to volume conduction effects (see Figures 4B to 4D). Similar results were obtained during the stimulus period; the only exception was the gamma band for which the coherence peak remained for all reference schemes (see supplementary Figure 4; refer to Srinath & Ray, 2014, for a more detailed discussion). Phase coherence was also high for some distance ranges such as 0.2√2 and 0.4 mm for bipolar reference and 0.4 mm for CSD, but this was simply an artifact of having part of the same signal during referencing. For example, if the single-wire referenced signals for two electrodes separated by 0.4 mm are V_1_ and V_2_, CSD_1_ will have a V_1_-V_2_/4 term while CSD2 will have a V_2_-V_1_/4 term, and this common component will lead to a spuriously high coherence and a phase difference of *π*. This effect is best illustrated by considering bipolar pairs separated by 0.4 mm, for which some pairs receive a contribution from a shared electrode (e.g., for three consecutive electrodes V1, V2, and V3 in a line, the bipolar pair V1-V2 and V2-V3) while others do not (for four electrodes V1 to V4 at vertices of a 0.4 mm × 0.4 mm square, bipolar pair V1-V2 and V3-V4). Phase coherence was high only for bipolar pairs with a shared electrode (see Figure 4C).

Coherence is a measure of phase consistency across time epochs, but it does not depend on the actual phase difference between the two signals. However, while averaging signals to compute the reference, the magnitude of the phase difference across electrode pairs is an important factor. For example, two signals that are perfectly out of phase have a coherence of 1 but will cancel each other out perfectly. We therefore studied the mean and variability of phase differences across electrodes as a function of frequency and interelectrode distance.

**Figure 5:**
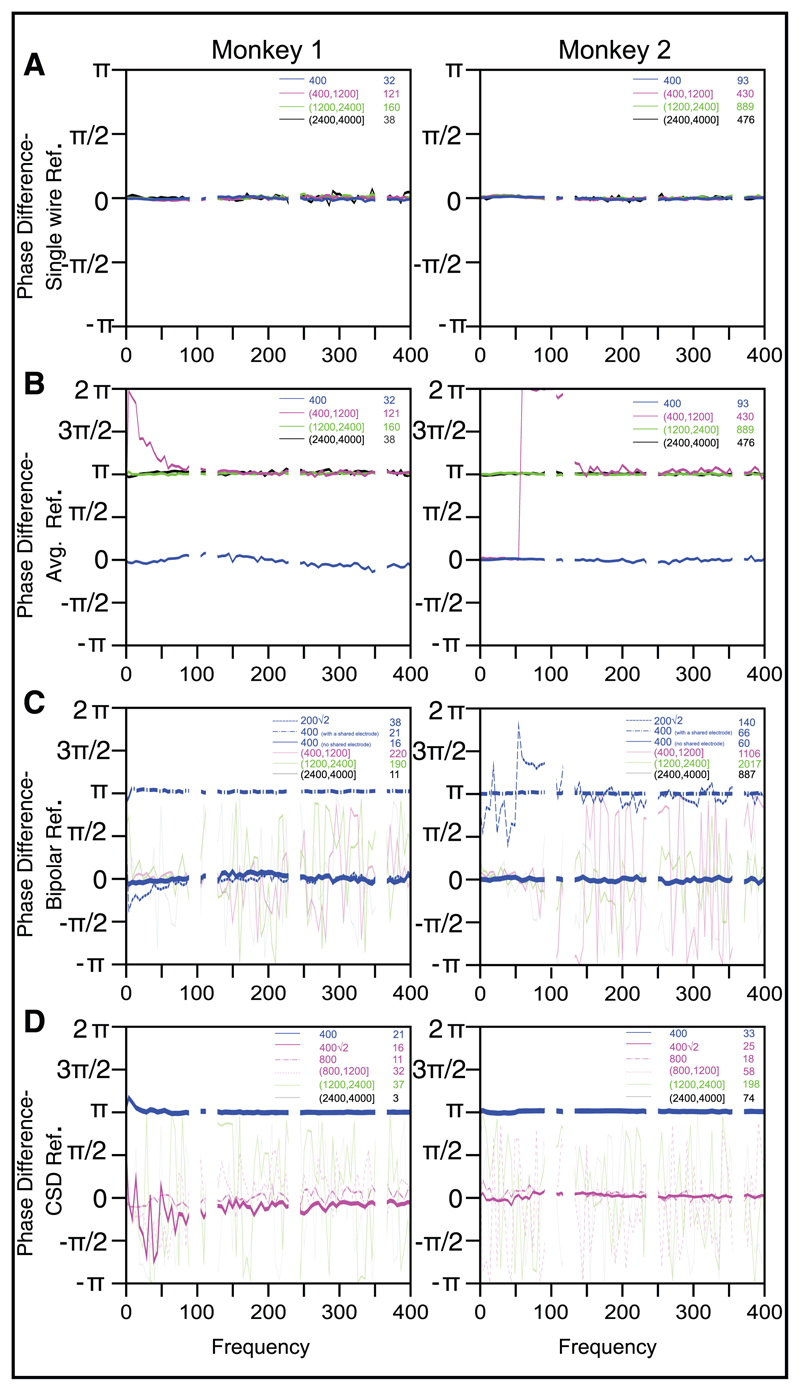
Mean phase difference between electrode pairs, averaged across all the electrode pairs within a distance range. Same format and analysis interval as Figure 4. For the bipolar reference scheme, the distance ranges for which the referenced signals share a common component (0.2√2 and 0.4 mm) are shown in dashed and dashed-dotted blue lines. Distance range 0.4 mm with no shared common component is shown as a thicker solid blue line. For the CSD reference scheme, the distance ranges for which the referenced signals share a common component (0.4, 0.4√2 and 0.8 mm) are shown in thicker lines. For the bipolar reference, the phase difference due to the deterministic component could be either 0 or *π* depending on how the signal is referenced (e.g., if the voltages recorded from three nearby electrodes are V1, V2, and V3, the bipolar referenced signals could be BP1 = V1-V2 and BP2 = V2-V3, which would produce a phase difference of *π*; or it could be BP1 = V1-V2 and BP2 = V3-V2, which would produce a phase difference of 0). For CSD, the phase difference at 0.4 mm is *π* because CSD at electrode 1 has a V1-V2/4 term while CSD at electrode 2 has a V2-V1/4 term. Electrodes separated by 0.4√2 and 0.8 mm share two or one neighbors, respectively, so their CSDs have a common component that leads to a phase difference of 0. If we ignore these special cases, phase differences for the remaining electrode pairs show a random value (thin lines in C and D) and high circular standard deviation (Figures 6C and D), suggesting that phase differences are random.

**Figure 6:**
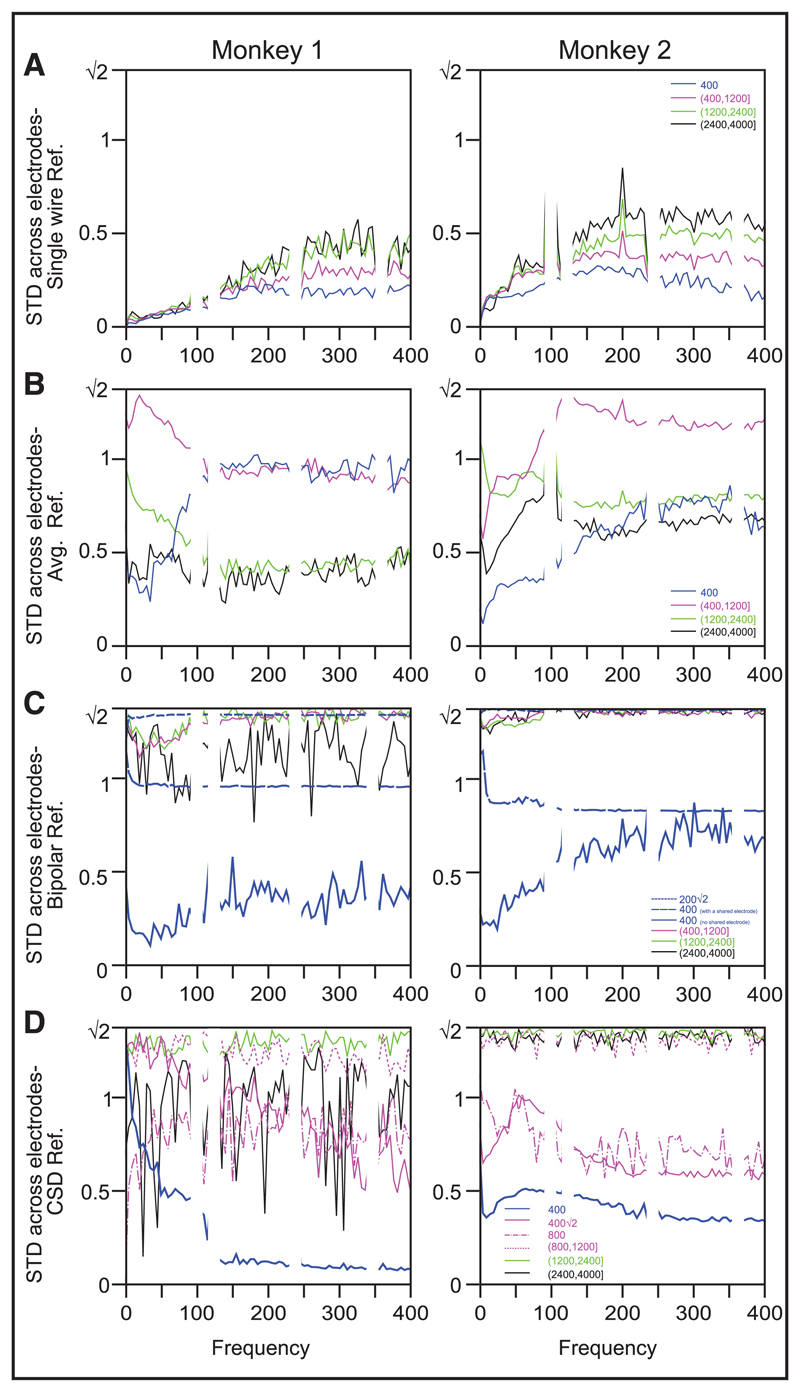
Circular standard deviation of phase differences across electrodes for different reference schemes. This measure has a range of 0 to √2. It uses the same format and analysis interval as Figure 5.

For the single-wire reference, the mean phase difference (across electrodes) was close to zero and was invariant of frequency or interelectrode distance (see Figure 5A), while the circular standard deviation (see Figure 6A) increased with frequency as well as interelectrode distance. Between 0 to 200 Hz, where the PSD slopes (see Figure 3A) and coherence (see Figure 4A) fell drastically, the circular standard deviation across electrodes was reasonably low (< 0.25 for nearby electrodes), so the signals from two electrodes were approximately in phase. Note that even at high frequencies, the mean phase difference remained zero throughout (instead of taking random values between −*π* and *π*), and the circular standard deviation was not close to √2, suggesting that the phases were not completely random across electrodes. Similar results were obtained by taking the mean of the absolute value of phase differences (not shown here), which is sometimes used to remove the ambiguity regarding the choice of the electrode position in a pair while computing the difference (i.e., given two electrodes, we could either use *φ*_1_ − *φ*_2_ or *φ*_2_ − *φ*_1_ as the phase difference).

These phase relationships provide a simple explanation of the effect of referencing. At low frequencies, signals from different electrodes that are averaged to get the reference signal have approximately the same phase and therefore do not cancel out with averaging, such that the low-frequency component of the reference signal is almost as large as the recording signal (see supplementary Figure 3) and subtracting the reference signal decreases the power at low frequencies appreciably and makes the slopes flatter as compared to the single-wire referenced signal. This effect is stronger for monkey 1 (see Figure 3A) because of higher phase coherence at low frequencies compared to monkey 2 (see Figure 4A), which resulted in a larger reference signal. The similarity between the reference signal and the recorded signal increases in the order of average reference, bipolar reference, and CSD, leading to more reduction in power and more flattening of the slope in the same order. At high frequencies beyond 200 Hz, all signals used for referencing have almost random phase, and therefore the reference signal is much weaker than the original single-wire signal (see supplementary Figure 3), so subtracting this reference does not affect the power or the slope.

The upward shift in the PSD at high frequencies for the bipolar reference (the blue trace is above the red and green traces in Figure 3A) can similarly be explained based on the phase relationships described above. When two sinusoids of the same frequency (*ω*) but different amplitudes √ (A_1_ and A_2_) and phases (*θ*_1_ and *θ*_2_) are added, the resulting signal is a sinusoid of frequency *ω* and amplitude √ (A_1_^2^+A_2_^2^ + 2 A_1_A_2_ cos (*θ*_1_–*θ*_2_)). So when amplitudes are equal and phases are random, the expected value of cos(*θ*_1_ − *θ*_2_) is zero and the amplitude is ~√2 times the original amplitude (whether two signals are added or subtracted does not make any difference because the phases are random). This explains why the power at higher frequencies increases by a factor of two with respect to the original signal (upward shift of the log PSD by log _10_(2), or about 0.3; blue trace in Figure 3A).

### 3.5 Effect of Referencing Scheme on Phase Differences

**Figure 7:**
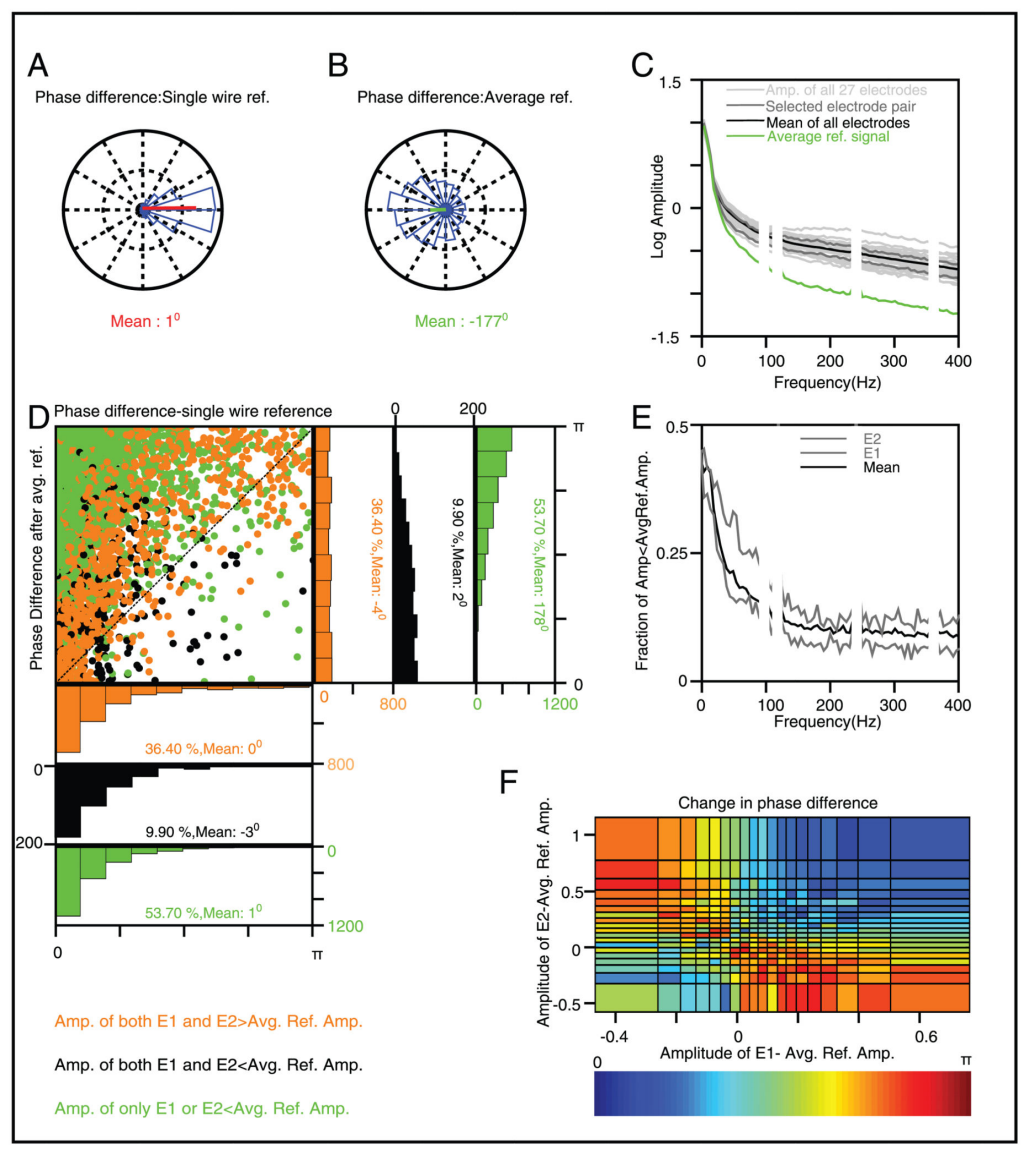
Effect of average referencing on the phase difference between a pair of electrodes separated by approximately 1.65 mm, recorded from the baseline period of monkey 1 (300–100 ms interval before stimulus onset). (A) Distribution of single-wire referenced phase differences in the 5–25 Hz frequency range. (B) Distribution of phase differences for the same two electrodes but after the signals are average referenced. (C) Amplitude spectrum of individual electrodes (light gray), electrode pair selected for analysis (dark gray), the average amplitude spectrum of all the electrodes (black), and the average reference signal (green). (D) Scatter plot and the corresponding histograms of the absolute phase differences in the 5–25 Hz frequency range, separated based on the three possible relationships between the amplitudes of two electrode amplitudes and the average reference amplitude. The mean phase differences and the corresponding percentage of trials are shown in the respective insets. (E) Fraction of trials for which the signal amplitude was less than the average reference amplitude for the two electrodes (gray) and the mean of the fractions of all the electrodes (black). (F) Change in absolute phase difference after average referencing as a function of the amplitudes for the two electrodes minus the average reference amplitude.

**Figure 8:**
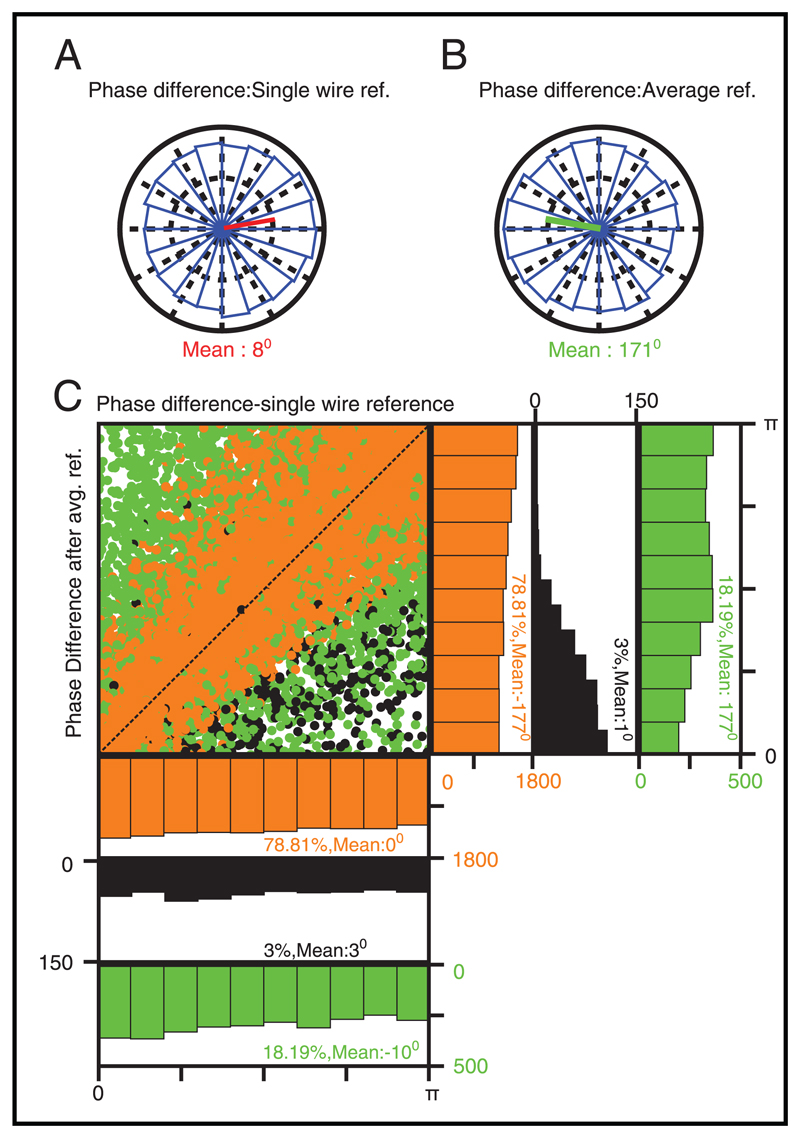
Same format and analysis interval as Figures 7A to 7C but for the frequency range of 200 Hz to 300 Hz. The mean vectors shown in panels A (in red) and B (in green) are lengthened by a factor of 10 for better clarity.

If the phase differences across electrodes for the single-wire reference are shown in a circular histogram, the distribution is skewed toward zero degrees at all frequencies (see, for example, Figure 7A; the degree of skewness might depend on the neural activity picked up by the reference wire and its proximity to the microelectrode grid), although the skewness decreases with increasing frequency as the circular standard deviation increases (see Figure 8A; the distribution becomes more spherical). Because all three referencing schemes essentially remove part of the common component present in the single-wire referenced signals, we expected the phase difference distributions to be more circular at all frequencies, with a mean phase difference of either zero (if the distribution is not perfectly circular) or random (if the distribution became completely circular) and an increase in the circular standard deviation in all cases. This was indeed observed for both bipolar and CSD references (see Figures 5C, 5D, 6C, and 6D)—apart from the distances for which there was a deterministic common component in the referenced signals that largely determined the phase difference (0.2√2 and 0.4 mm, with a shared electrode, for bipolar; 0.4, 0.4√2, and 0.8 for CSD), the phase differences were distributed randomly, and the CSD was close to √2. However, the results obtained for the average reference signal were counterintuitive. While the mean phase differences remained close to zero at 0.4 mm (which was expected, because the coherence did not decrease to baseline levels for this distance range—see Figure 4B—suggesting that some of the common component in the signals remained even after referencing), the mean phase differences shifted to *π* at high frequencies for the distance range between 0.4 mm and 1.2 mm and all frequencies for larger distances (see Figure 5B). In terms of the polar plot, this suggests that although the reference signal was much smaller than the original signal (especially at high frequencies; see supplementary Figure 3), subtracting this small signal and again computing the phase differences caused the skew to shift from zero to *π* (see Figures 7B and 8B). The CSD also showed a nonintuitive trend: as a whole, it increased as compared to the single-wire reference (see Figure 6B versus 6A), but it was higher for intermediate distances (0.4–1.2 mm) and reduced at higher distance ranges (>1.2 mm).

To explain this effect, we took a representative pair of electrodes separated by approximately 1.65 mm (√(1.6^2^ + 0.4^2^)) and studied how the phase differences changed once the signals were average referenced. Figures 7A and 7B show the circular histogram of the mean phase difference for frequencies between 5 Hz and 25 Hz (the results were similar when a different low-frequency range was selected) for single-wire and average reference schemes. Consistent with the results shown in Figure 5, the mean phase difference shifted from approximately 0 degrees for single-wire reference to approximately 180 degrees for average reference. A similar trend was observed at high frequencies (200–300 Hz; results remain the same for a different frequency range; see Figures 8A and 8B). This was counterintuitive because the amplitude of the average reference signal (obtained by averaging the single-wire referenced electrodes) was smaller than any of the electrodes, especially at high frequencies (see the green trace in Figure 7C), and therefore subtraction of this small average reference signal from individual electrode was not expected to change the phases substantially.

There are two reasons that average referencing produces the observed phase shift. The first reason, which is more applicable at low frequencies, is related to the high variability of the spectral estimator used to calculate the amplitude spectrum (see Jarvis & Mitra, 2001; Srinath & Ray, 2014). In our data, because we used a single taper to estimate the PSD, the power followed an exponential distribution across trials, while the amplitude followed a Rayleigh distribution (see Srinath & Ray, 2014). Rayleigh distribution has a significant proportion of values that are very small, so for a given trial, no matter how small the average reference signal was, there were always some electrodes for which the signal amplitude fell below the average reference amplitude, especially at low frequencies. To demonstrate this, we plotted the fraction of trials for which the signal amplitude was a smaller-than-average reference amplitude (see Figure 7E). The fraction expectedly decreased with increasing frequency, but even at high frequency, when the mean amplitude (across trials) of either electrode was larger than the mean amplitude of the average reference signal by an order of magnitude (Figure 7C), in about 10% of the trials, the average reference amplitude was larger than the signal amplitude. This proportion was almost 40% at low frequencies because the coherence was high and the average reference signal was relatively much larger.

At low frequencies, phase differences were small across electrode pairs in a single-wire reference scheme, so if we represent the amplitudes and phases of individual electrodes as vectors, all signal vectors, as well as the vector corresponding to the average reference signal, would point in approximately the same direction. Subtracting this average reference signal would keep the phase approximately the same if the signal amplitude was larger than the average reference amplitude; otherwise the vector should show a shift of *π*. Therefore, assuming the original phase difference to be zero, the final phase difference would be *π* if exactly one of the two electrodes had an amplitude greater than the average reference amplitude.

To illustrate this, we plotted the absolute phase difference in the single-wire scheme versus the absolute phase difference after average referencing (see Figure 7D). We separated the trials into three categories: when both (orange), exactly one of the two (green), or neither of the two (black) signal amplitudes was larger than the average reference amplitude. For trials in which either both amplitudes (orange) or neither (black) were larger, the mean phase difference remained close to zero degrees even after average referencing, although the distribution was much less skewed (larger circular standard deviation). However, when only one amplitude was larger than the average reference (green), the mean phase difference indeed shifted to *π*. Overall, the mean circular distribution became more spherical (increase in standard deviation) after average referencing (see Figure 7B), but the large shift of *π* in the phase difference of selected trials caused the overall phase difference to have a slight skew toward *π*. This effect was further illustrated by plotting the mean absolute shift in phase difference due to referencing as a function of the amplitudes of the two signals after subtracting the average reference amplitude (see Figure 7F). In this plot, the first quadrant corresponds to the trials shown in orange in Figure 7D, the third quadrant corresponds to black, and the remaining quadrants correspond to green trials. Indeed, the mean absolute phase shift was about 0 for orange and black trials and *π* for the green trials.

At high frequencies (200–300 Hz), the overall phase differences were much more scattered (see Figure 8A). Even for this case, there was a large shift in the mean phase after average referencing (see Figure 8B). The reason described for the low-frequency case is insufficient to explain this result because now the average reference signal was very small, and consequently, there were fewer trials for which either one or both electrodes had amplitude less than the average reference. However, we noticed another trend in this case: even for the trials for which both electrodes had amplitude greater than the average reference, the distribution shifted toward *π*, even though none of the trials showed a large change in phase difference when average referenced (see Figure 8C, orange dots).

This behavior can be explained based on vector algebra. First, without loss of generality, assume that the phase of the average reference signal is 0 degrees, such that average referencing involves adding a small vector pointing toward *π*. Also, assume that the magnitude of the signal is larger than the average reference. Applying average reference to a signal shifts the phase toward *π*, but the magnitude of the shift depends on the signal phase. If an electrode has a phase close to zero, it would not change by much after average referencing (it would shift by *π* for a small fraction of trials for which the signal amplitude is less than the average reference amplitude, as discussed in Figure 7). But if the signal phase is close to *π*/2, the signal vector would move substantially toward *π* after average referencing. This explains why the scatter in phase difference after average reference increases with increasing phase difference for the single-wire reference: if two electrodes have a very small phase difference in the single-wire reference (phase difference close to zero), both phases shift by approximately the same amount, and therefore the phase difference would remain small after average referencing. However, if the phase difference is large, the phase would shift by dissimilar amounts after average referencing, and the resulting phase difference would be more scattered around the single-wire phase difference values.

Now consider the case when the phase difference between electrodes is close to *π*/2. This could happen if the first phase is anywhere between zero and *π*/2, while the second is shifted further by *π*/2. When the first phase is close to zero and second is close to *π*/2, applying average reference would leave the first phase unchanged, but the second one would move toward *π*, and therefore the overall phase difference would increase. This would happen as long as the first phase is less than *π*/4. The opposite would be observed if the first phase is near *π*/2 and the second near *π*; now the first one would shift towards *π* and the second would not change much, and therefore the phase difference would decrease after applying the average reference. However, the percentage of trials for which this happens would be fewer than the earlier case, because these signal vectors (along with other electrodes) are averaged to get the average reference vector and therefore are more likely to be pointing toward the average reference vector. Overall, this would cause an asymmetric shift in the phase differences, with an overall upward shift in the orange dots in the middle portion of the plot. Indeed, we observed that more dots were above the diagonal than below, and therefore the distribution as a whole shifted toward *π*. Also, as the interelectrode distance increased, average referencing changed the skew in the phase histogram from 0 to *π*. The histogram was more circular at intermediate distances, due to which the circular standard deviation was larger (see Figure 6B).

## 4 Discussion

We studied the effect of stimulus, behavior, and referencing on power and phase of LFP signals recorded using microelectrode arrays from V1 cortex of awake monkeys. The signals were originally recorded with reference to a single wire on the dura of the monkeys (single-wire reference), and were re-referenced using three popular schemes: average reference, bipolar, and CSD (see section 2 for details). We found that the power of the LFP signal and the slope of the PSD depended on the reference scheme at low frequencies (< 200 Hz) but became invariant at higher frequencies. These results were explained based on the coherence profile across electrode pairs, which was high at low frequency for the single-wire reference but decreased to baseline levels at higher frequencies. Further, the coherence decreased to baseline levels at all frequencies for other reference schemes, suggesting that the low-frequency coherence of the single-wire reference signals was due to a common source, whose contribution was removed by referencing leading to a decrease in PSD power and slope. Most important, we found that average reference caused the mean phase difference across electrodes to shift from zero to *π*. This was due to two reasons. First, due to the variability of the spectral estimator, a fraction of electrodes always had a magnitude less than the average reference signal on any given trial, such that a fraction of phase differences shifted from 0 to *π* with average referencing (see Figure 7; more applicable at low frequencies). Second, phases shifted by different amounts with average referencing that depended on the signal phase relative to the average reference phase (see the orange dots in Figure 8C; see section 3 for details). Varying stimulus contrast or attentional focus changed the PSDs at alpha and gamma bands, but otherwise had little effect on the PSD slopes or phase differences.

### 4.1 Reference Techniques and Their Uses

In EEG or ECoG recordings, the use of single-wire reference induces biases in phase coherence across sites, which depend on the amplitude of the reference signal itself (Fein et al., 1988; Hu et al., 2007, Hu, Stead, Dai, & Worrell, 2010; Nunez & Srinivasan, 2006; Schiff, 2005). Different reference techniques have traditionally been explored and used in the context of EEG recordings, each with its advantages as well as shortcomings (Bertrand, Perrin, & Pernier, 1985; Qin, Xu, & Yao, 2010; Dien, 1998; Nunez et al., 1997). With the advent of microelectrode recordings, these reference techniques can be used similarly for LFP recordings as well. Average referencing is used to remove any external noise that is common to all the sites and is believed to yield better signal-to-noise ratios (Crone, Boatman, Gordon, & Hao, 2001; Yuval-Greenberg, Tomer, Keren, Nelken, & Deouell, 2008; Ludwig et al., 2009; Sinai et al., 2009; Boatman-Reich et al., 2010; Podvalny et al., 2015). Bipolar referencing is preferred if one wants to magnify highly local events that occur near an electrode (DeCoteau et al., 2007; Hamamé et al., 2014; Bosman et al., 2012; Brunet et al., 2014; Spaak, Bonnefond, Maier, Leopold, & Jensen, 2012; van Kerkoerle et al., 2014). For example, van Kerkoerle and colleagues (2014) showed that the interarea (V1-V4) coherence profile depended on whether a global (similar to single-wire reference here) or a local reference (similar to bipolar reference here) was chosen (see their Figure 7B). In particular, the overall coherence across all frequencies dropped drastically when bipolar reference was chosen, but a frequency dependence also emerged (the gamma band showed relatively higher coherence than other frequencies), which was not seen very saliently earlier. CSD analysis has been used in the case of extracellular recordings to extract local effects in the form of current sources and sinks (Einevoll et al., 2013; Lakatos, Chen, O’Connell, Mills, & Schroeder, 2007; Lakatos, Karmos, Mehta, Ulbert, & Schroeder, 2008; Schroeder et al., 2001). These different referencing techniques have been used across recording modalities, at varying scales of spatial resolution, to evaluate properties of signals such as the spectral characteristics and phase coupling (or coherency) (Bosman et al., 2012; Brunet et al., 2015; 2014; Ng, Logothetis, & Kayser, 2013; Lachaux et al., 1999) or cross-frequency phase-amplitude coupling (He, 2014; He, Zempel, Snyder, & Raichle, 2010). However, how these referencing techniques might themselves affect the PSDs and the phase relationship between LFPs at different recording sites has not been evaluated. Here we show that these features of the signal are indeed sensitive to the reference technique. In particular, our results show that average reference should be avoided while doing phase analysis. While computing PSD slopes, it is better, wherever possible, to focus on a frequency range for which different reference schemes give similar results (> 200 Hz), although most LFP analyses are typically limited to approximately 200 Hz and PSDs in different frequency ranges can have different well-characterized slopes (Bedard et al., 2006a). Another possibility is to use more advanced methods to estimate the reference signal (which is subsequently used for re-referencing), such as reference estimation standardization technique (REST) or robust maximum likelihood type estimation (see Lepage, Kramer, & Chu, 2014, for details).

### 4.2 Spectral Slopes and Underlying Mechanisms

Consistent with our results, several reports have shown PSD slopes between 1 and 3 at frequency ranges below 100 Hz in both EEG and ECoG (Dehghani, Bédard, Cash, Halgren, & Destexhe, 2010; Freeman, Holmes, West, & Vanhatalo, 2006; Freeman, Rogers, Holmes, & Silbergeld, 2000; He et al., 2010; Miller et al., 2009; Podvalny et al., 2015; Pritchard, 1992) as well as LFP (Bédard et al., 2006a; Petermann et al., 2009). Slopes in this frequency range critically depend on the referencing scheme, so these results must be interpreted with caution.

Although studies involving LFP and ECoG have traditionally focused on oscillations below about 100 Hz, recent studies have shown the existence of both fast oscillations (Buzsaki & Draguhn, 2004; Scheffer-Teixeira, Belchior, Leão, Ribeiro, & Tort, 2013) and asynchronous “broadband” activity at frequencies above about 100 Hz (Manning, Jacobs, Fried, & Kahana, 2009; Miller et al., 2009; Ray & Maunsell, 2011a). However, fewer reports have studied the PSD slopes at frequencies above about 100 Hz. Milstein and colleagues (2009) recorded LFP from humans using intracranial electrodes (40 *μ*m microwires) and reported a slope of about 2 between 1 Hz and 400 Hz, which they related to slow dendro-synaptic decay or up-down state changes characteristic of slow wave sleep (telegraph noise). Miller and colleagues (2009) recorded ECoG data from humans and reported a slope of 4 between approximately 80 Hz and 500 Hz. These results are inconsistent with our findings (slopes of about 1.4 above 200 Hz). One reason for this difference could be the difference in species (monkey versus human) or the size of electrodes (microelectrode versus ECoG electrodes). Another reason could be the fast stimulus presentation rates and short analysis periods used in our study. We used a brief temporal window of up to 500 ms for computing PSDs, while most of the other reports have used data spanning seconds to minutes with either no task or passive fixation. For example, the slope of 2 observed by Milstein and colleagues (2009) was due to the alternation of up and down states due to slow waves, but that possibility cannot be tested in our data due to the fast stimulus presentation times during which the monkeys performing our task had to constantly be in an attentive state and had short recovery periods between stimuli. This could also explain why our different stimulus or behavioral conditions did not change the PSDs except at alpha and gamma ranges, unlike previous studies that have shown that slopes generally tend to decrease on stimulus presentation (Podvalny et al., 2015) or as one goes from a state of rest to elevated levels of alertness or wakefulness as demanded by the task and cognitive load (see He, 2014, for a discussion).

A careful characterization of PSD slope is essential because it reveals properties of the underlying network. For example, a slope of approximately 2, found in many studies (see above), can arise from Brownian noise, and several mechanisms have been suggested that could generate this noise. Some of the mechanisms explored in modeling studies and attributed to the observed PSD slopes are temporal dynamics of the synaptic processes (fast rise and slow decay exponentially) triggered by Poisson spiking (Milstein et al., 2009; Bédard et al., 2006a; Freeman & Zhai, 2009), dendritic filtering properties (Miller et al., 2009; Pettersen & Einevoll, 2008; Lindé et al., 2010), ionic diffusion processes across the membrane, and filtering properties of extracellular medium (Bédard, Kröger, & Destexhe, 2004; Bédard et al., 2006b; Bédard & Destexhe, 2009; Logothetis et al., 2007). In this study, since the PSD slopes are observed to depend critically on the reference scheme and stimulus condition at low frequencies, it is difficult to infer the noise or filtering properties of the network using LFP data. However, at higher frequencies, the slope was invariant to reference scheme or whether a stimulus was presented. It was about 1.4 for the two monkeys, inconsistent with the mechanisms described in the studies mentioned above that produce an integer value of the PSD slope (about 2 for Milstein et al., 2009, and 3 for Bédard et al., 2006a). This is instead indicative of a fractal (self-organized critical or SOC) behavior (Bak, Tang, & Wiesenfeld, 1987, 1988; Beggs & Plenz, 2003; Dehghani et al., 2012), although simply showing a power law form in the PSD does not guarantee SOC behavior (for a detailed review on this topic, see Beggs & Timme, 2012). Finally, PSD slopes of signals like ECoG and EEG could potentially be used to infer abnormalities in the underlying network activity in pathological conditions such as schizophrenia and autism spectrum disorders (Voytek & Knight, 2015).

Although the PSD slopes did not change with increasing stimulus contrast, the power increased over a broad frequency range leading to an upward shift of the PSD, similar to previous studies that have related this “broadband shift” to an increase in firing rates of neurons near the microelectrode (Manning et al., 2009; Ray, Crone, Niebur, Franaszczuk, & Hsiao, 2008; Ray & Maunsell, 2011a). To accurately estimate this broadband power increase, we need to discount the changes in power in narrow-band oscillations such as delta, theta, alpha, beta, and gamma, which was achieved by Manning and colleagues (2009) by using a robust regression fit instead of a least squares fit. In their case, data were recorded from many different brain areas where different oscillations were prevalent, which necessitated their approach. In our case, this was not required because data were recorded from primary visual cortex, where only an alpha peak was observed in the PSD. Calculation of slopes between 0 Hz to 150 Hz after ignoring the power in classical frequency bands (similar to the approach used by Manning and colleagues) yielded similar results (data not shown).

### 4.3 Significance of Absolute Phases

In most reports, the absolute signal phase or phase difference between two electrodes is computed using any one referencing scheme and the change in phase across stimulus or behavioral conditions is studied. Had the change in phase with stimulus/behavior been the only important factor, the shift in phase by 180 degrees due to average referencing would not have mattered because the change would have remained the same. However, there are many cases in which the absolute phase or phase difference between electrodes is mapped to physiological properties. For example, the communication through coherence (CTC) hypothesis (Fries, 2005) proposes that if two neuronal assemblies oscillate with a phase difference equal to the conduction delay (of spikes) between them, spikes produced at the most excitable phase of the first assembly reach precisely when the second assembly is most excitable, so that the second assembly can fire as well. If the phase difference is not at the optimum value, the second assembly does not produce a spike, thereby allowing flexible long-range communication between neuronal assemblies depending on the magnitude and consistency of the phase differences (Fries, 2005; Schoffelen, Oostenveld, & Fries, 2005; Womelsdorf et al., 2007; Gregoriou, Gotts, Zhou, & Desimone, 2009; Bosman et al., 2012; Roberts et al., 2013; Jia, Tanabe, & Kohn, 2013). Here the phase difference is mapped to the efficacy of communication channels: zero degrees implies efficient communication, while 180 degrees implies poor communication, and the results get completely flipped if average referencing is used instead of single-wire reference. Phase differences are also sometimes used to determine the direction of information transmission between two brain areas (van Kerkoerle et al., 2014; see their Figure 7C), which would get reversed if average reference is used (these authors used single-wire or bipolar reference).

Similarly, some hypotheses make specific predictions about the absolute phase of an oscillation with spiking activity or perceptual or attentional state. For example, in theta/gamma phase coding hypothesis (Buzsaki & Chrobak, 1995; Fries, Nikolić, & Singer, 2007), the phase of the signal is mapped to the level of inhibition in the network (which is maximum at the peak of the oscillation and minimum at the trough), such that the position of the spike with respect to the oscillation can be used to code the strength of the stimulus. Average referencing flips the phase by 180 degrees whenever the signal amplitude is less than the reference signal, which happens for a large proportion of trials at low frequencies (see Figure 7E). For such trials, if the spike actually occurs at the trough of the signal, after average referencing, it would appear at the peak instead, completely changing the results. Similarly, several studies have reported that perceptual threshold or attentional state depends on the phase of theta or alpha oscillations (Ai & Ro, 2014; Busch, Dubois, & Van Rullen, 2009; Busch & VanRullen, 2010; Jensen, Gips, Bergmann, & Bonnefond, 2014; Mathewson, Gratton, Fabiani, Beck, & Ro, 2009). Again, using the average referencing scheme would change the phase of a subset of trials by 180 degrees, leading to an incorrect interpretation. This would happen even if the same reference scheme were used for all the behavioral conditions, because the hypothesis posits a specific relationship between behavior and the absolute phase of the signal.

In recent years, there has been an increase in LFP recordings with several microelectrodes, a critical step toward understanding the network properties at a finer scale and studying connectivity, communication, and information transfer in small networks. Our results highlight the changes in power and phase relationship due to different reference schemes, which must be properly accounted for before using this information to gain insights into the network.

## Supplementary Material

Supplementary figures
