# Supplementary material for: Effect of Reference Scheme on Power and Phase of the Local Field Potential
Source: Neural Comput. Author manuscript; Available in PMC 2020 Apr 2. (PMC7117962; doi:10.1162/NECO_a_00827)
Supplement: Supplementary figures [file EMS86144-supplement-Supplementary_figures.pdf]

Supplementary Figure 1: PSD amplifier roll-off correction and slope calculation details. The transfer function of the Blackrock data acquisition is determined experimentally by passing a sinusoidal signal at various frequencies and measuring the output. Black dots show the power (square of the amplitude) of the transfer function, which remains flat until ~400 Hz. The transfer function power interpolated from the data is shown in red, while theoretical transfer function power of the 4th order Low Pass Butterworth filter (with a cutoff of 500 Hz, as used the Blackrock data acquisition system) is shown by the dotted cyan line. A) Average Raw PSD of 27 electrodes obtained during the baseline period (500 to 0 ms before stimulus onset) for Monkey 1 is shown in gray. The same PSD after amplifier transfer function correction, done by subtracting the red line from the gray ($\log_{10}PSD = \log_{10}PSD_{Raw} - \log_{10}(Power_{TF}) = \log_{10}(PSD_{Raw}/Power_{TF})$) is shown in black (same trace is shown in Figure 1 on a linear frequency axis). When the noise floor is negligible (which is the case in our data), equation 4 reduces to a straight line on a log-log scale ($\log P = \log(A) - \alpha\log(f)$). Results of curve fits on 30 Hz segments centered at 50, 150 and 300 Hz and their slope values are shown in blue, green and orange, respectively (slope values for raw PSDs are shown in a lighter shade). B) Same as A, for 62 electrodes recorded from Monkey 2.

Supplementary Figure 2: A) Effect of the frequency range used to fit the curve for slope computation. PSD is computed over a 500 ms interval, which yields a frequency resolution of 2 Hz. Each curve represents the mean slope values calculated using different fit ranges as indicated in the inset. The shaded area around each line denotes the SEM. Longer ranges (50 Hz, 100 Hz) smoothen the curve and give spurious values in the low frequency range due to unavailability of enough data points on the lower side. B) Average PSD of the LFP using signal of length 200 ms (300 ms to 100 ms before stimulus onset across all stimulus conditions), 500 ms (500 ms to 0 ms before stimulus onset across all stimulus conditions) and 1000 ms (500 ms before stimulus onset to 500 ms after stimulus onset for the 0 % stimulus

contrast condition) are shown in cyan, red and mustard. N denotes the number of electrodes used for analyses. Power at frequencies around the monitor refresh rate and noise harmonics (± 8 Hz) has been masked to visualize the PSDs better. C) Slopes of the PSDs shown in B for a fit length of 30 Hz. Note that for the 200 ms condition, the frequency resolution was 5 Hz and only 7 points were available for curve fitting at each frequency. If the fit range included a noise peak (such as 240 Hz), that data point was removed, so the fit was only done on 6 data points. However, occasionally the noise peak was broader and increased the power at neighboring frequencies (235 and 245 Hz), which lead to a spuriously high slope value at frequencies just above that noise peak (250 and 260 Hz; since power at the left side were higher than usual). Similarly, a spuriously low value was obtained at some frequencies below the noise peak (230 Hz). This effect vanished when a longer interval was used because the frequency resolution was higher (2 Hz and 1 Hz for 500 and 1000 ms, respectively) and more data points were available in the 30 Hz fit range.

Supplementary Figure 3: PSD of the reference signals (that are subtracted from the single-wire referenced signal): bipolar (blue, whose mean PSD is same as that of the mean PSD of single-wire), CSD (orange), and average reference (green).

Supplementary Figure 4: Same as Figure 4, but the PSDs are computed between 200-400 ms after the onset of a Gabor stimulus at 100% contrast. Note that because we use a Gabor stimulus that does not cover the receptive fields of all the sites, this analysis includes electrodes that are not well stimulated. Here we cannot restrict the analysis to only well-stimulated sites (like Figure 2), because the average reference signal is constructed using all the electrodes. We therefore performed the same analysis on a different dataset in which gratings of various sizes were presented inside the receptive field while the monkey performed an orientation detection task on the opposite hemifield, and used the condition

when the largest grating was presented (same dataset as used in (Srinath and Ray 2014)) such that all the electrodes that were used for generating the reference signal were well stimulated. All the results shown here remain unchanged if this dataset was used instead.

Supplementary Figure 5: Same as Figure 5, but for the stimulus condition.

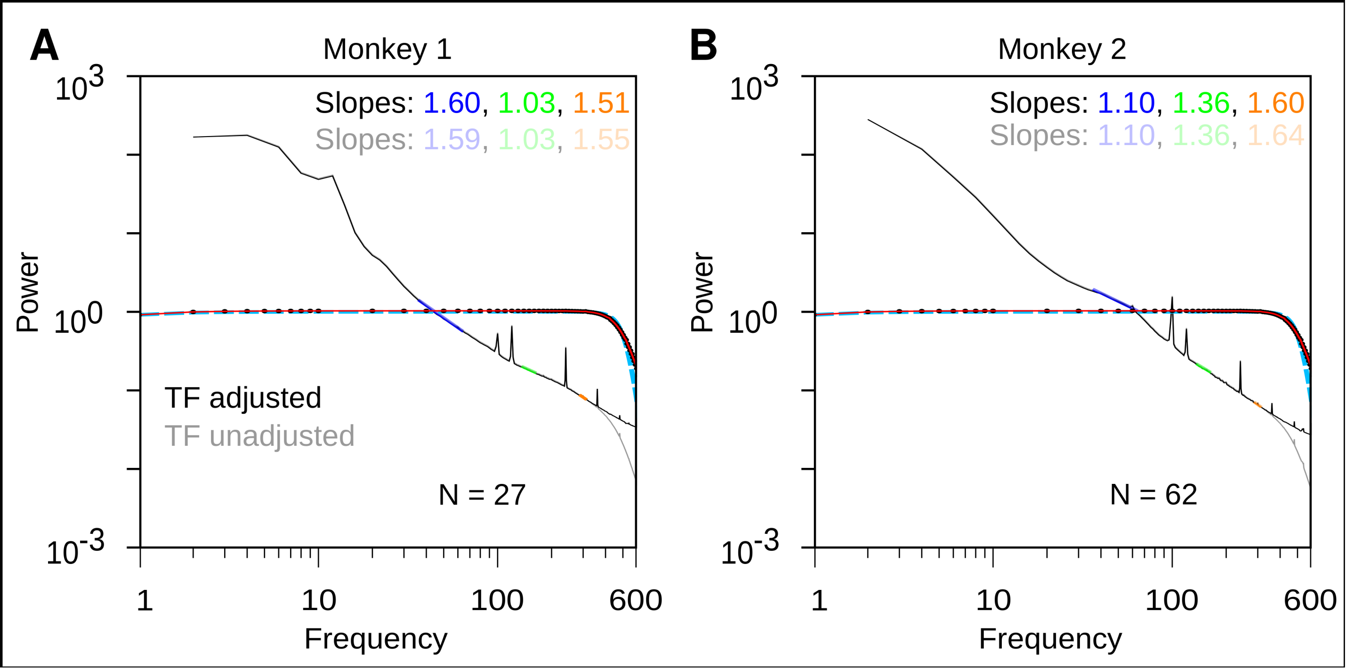
A
Monkey 1
Slopes: 1.60, 1.03, 1.51
Slopes: 1.59, 1.03, 1.55
TF adjusted
TF unadjusted
N = 27
Power
Frequency
B
Monkey 2
Slopes: 1.10, 1.36, 1.60
Slopes: 1.10, 1.36, 1.64
N = 62
Power
Frequency

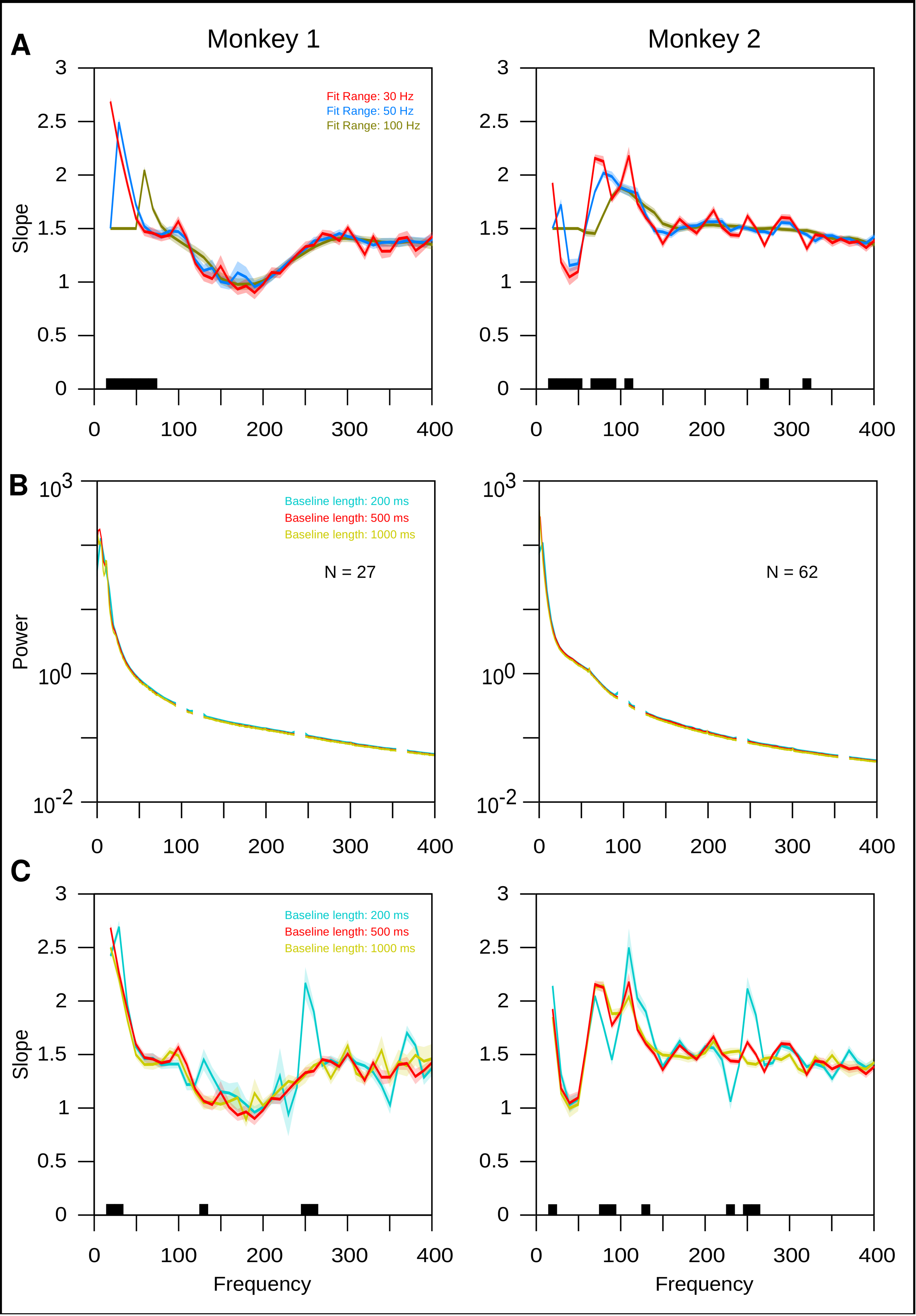

A
Monkey 1
Monkey 2
Fit Range: 30 Hz
Fit Range: 50 Hz
Fit Range: 100 Hz
Slope
B
Baseline length: 200 ms
Baseline length: 500 ms
Baseline length: 1000 ms
N = 27
N = 62
Power
C
Baseline length: 200 ms
Baseline length: 500 ms
Baseline length: 1000 ms
Slope
Frequency
Frequency

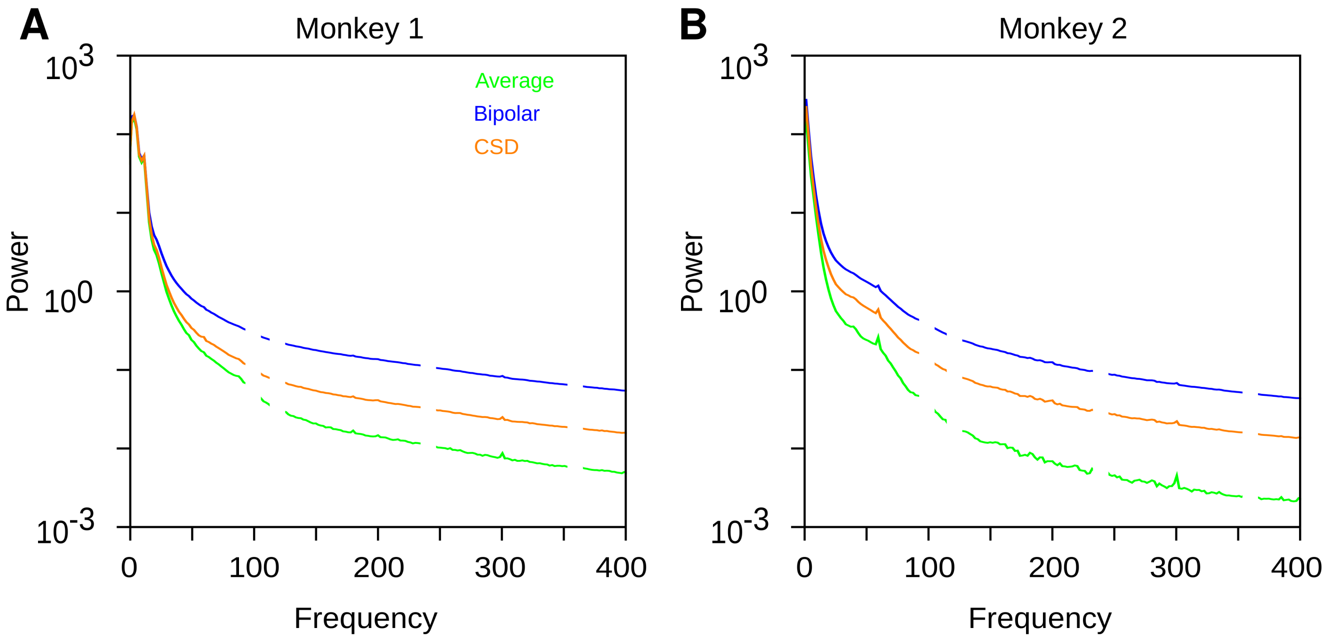

A
Monkey 1
B
Monkey 2
Average
Bipolar
CSD
Power
Frequency
10^3
10^0
10^{-3}
0
100
200
300
400

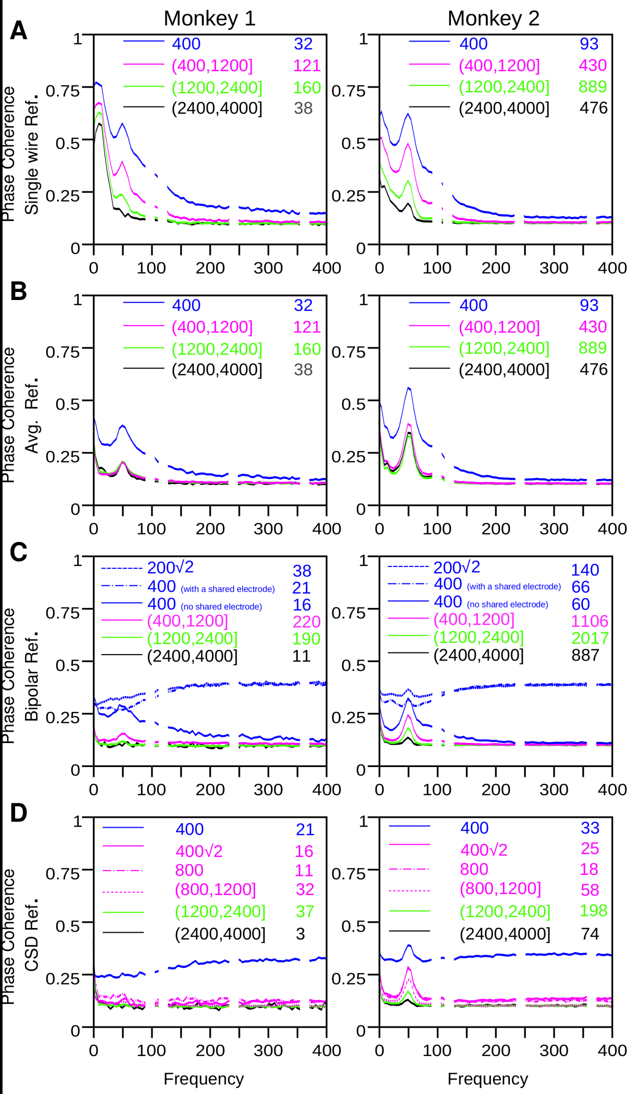
Monkey 1
Monkey 2
A
B
C
D
Phase Coherence Single wire Ref.
Phase Coherence Avg. Ref.
Phase Coherence Bipolar Ref.
Phase Coherence CSD Ref.
400 32
(400,1200] 121
(1200,2400] 160
(2400,4000] 38
400 93
(400,1200] 430
(1200,2400] 889
(2400,4000] 476
200√2 38
400 (with a shared electrode) 21
400 (no shared electrode) 16
(400,1200] 220
(1200,2400] 190
(2400,4000] 11
200√2 140
400 (with a shared electrode) 66
400 (no shared electrode) 60
(400,1200] 1106
(1200,2400] 2017
(2400,4000] 887
400 21
400√2 16
800 11
(800,1200] 32
(1200,2400] 37
(2400,4000] 3
400 33
400√2 25
800 18
(800,1200] 58
(1200,2400] 198
(2400,4000] 74
0
0.25
0.5
0.75
1
0
100
200
300
400
Frequency

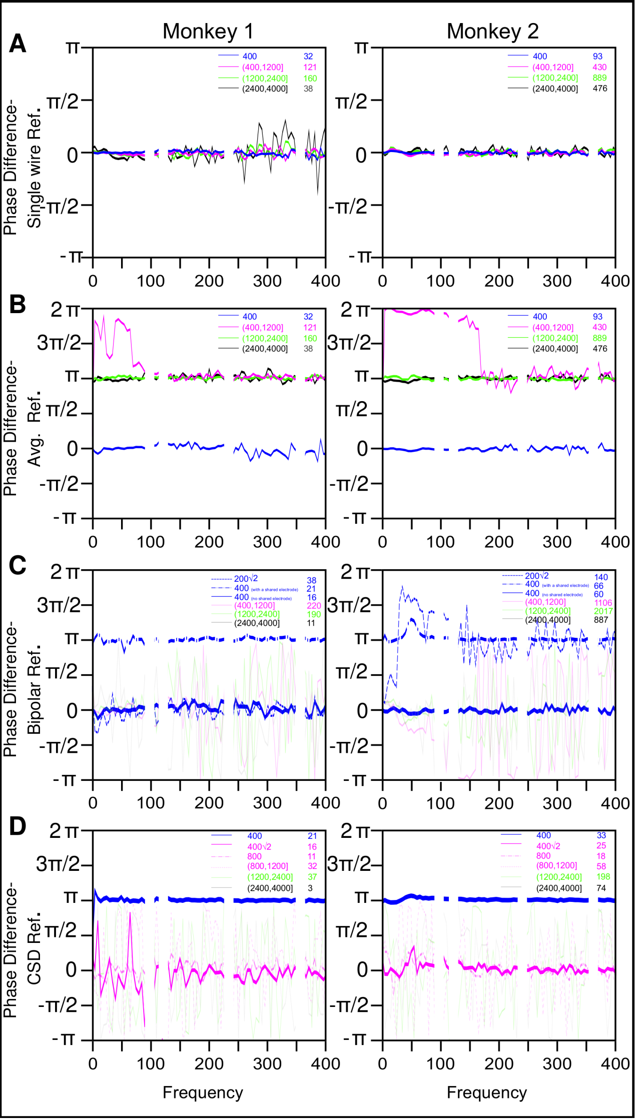

Monkey 1
Monkey 2
A
B
C
D
Phase Difference-
Single wire Ref.
Avg. Ref.
Bipolar Ref.
CSD Ref.
π
π/2
0
-π/2
- π
2 π
3π/2
0
100
200
300
400
400 32
(400,1200] 121
(1200,2400] 160
(2400,4000] 38
400 93
(400,1200] 430
(1200,2400] 889
(2400,4000] 476
200√2 38
400 (with a shared electrode) 21
400 (no shared electrode) 16
(400,1200] 220
(1200,2400] 190
(2400,4000] 11
200√2 140
400 (with a shared electrode) 66
400 (no shared electrode) 60
(400,1200] 1106
(1200,2400] 2017
(2400,4000] 887
400 21
400√2 16
800 11
(800,1200] 32
(1200,2400] 37
(2400,4000] 3
400 33
400√2 25
800 18
(800,1200] 58
(1200,2400] 198
(2400,4000] 74
Frequency
